# A Transferable “Resistance Factor” from in vitro Cultured MDMS-Resistant Yoshida Sarcoma Cells

**DOI:** 10.1038/bjc.1973.29

**Published:** 1973-03

**Authors:** B. Szende, M. Fox, B. W. Fox

## Abstract

Cells of the methylene dimethanesulphonate-(MDMS)-resistant Yoshida sarcoma cell line contain a low molecular weight “resistance factor” which is present in the culture medium of these cells and may be utilized by MDMS-sensitive Yoshida sarcoma cells either by co-culturing the two cell lines or by culturing the MDMS-sensitive Yoshida cells in a medium containing 20% used medium of MDMS-resistant Yoshida cells or in the presence of dialysed medium from resistant cells. The “resistance factor” does not inactivate the drug itself or its metabolites, and it has no influence on the sensitivity of the cells if added after MDMS treatment. Twenty-four hours seems to be enough time for the transfer of the resistance factor, but its effect on whole populations decreases within 24 hours of ceasing the supply. The relationship between these findings and the known phenomena of metabolic co-operation are discussed.


Br. J. Cancer (1973) 27, 245

### A TRANSFERABLE " RESISTANCE FACTOR " FROM *IN VITRO* CULTURED MDMS-RESISTANT YOSHIDA SARCOMA CELLS

B. SZENDE,* M. FOX AND B. W. FOX

*From the Paterson Laboratories, Christie Hospital and Holt Radium Institute, Manchester M20 9BX*

Received 22 November 1972. Accepted 15 December 1972

**Summary.—Cells of the methylene dimethanesulphonate-(MDMS)-resistant Yoshida sarcoma cell line contain a low molecular weight " resistance factor " which is present in the culture medium of these cells and may be utilized by MDMS-sensitive Yoshida sarcoma cells either by co-culturing the two cell lines or by culturing the MDMS-sensitive Yoshida cells in a medium containing 20% used medium of MDMS-resistant Yoshida cells or in the presence of dialysed medium from resistant cells. The " resistance factor " does not inactivate the drug itself or its metabolites, and it has no influence on the sensitivity of the cells if added after MDMS treatment. Twenty-four hours seems to be enough time for the transfer of the resistance factor, but its effect on whole populations decreases within 24 hours of ceasing the supply. The relationship between these findings and the known phenomena of metabolic co-operation are discussed.**

METABOLIC co-operation has been demonstrated by a number of workers (Subak-Sharpe, Bürk and Pitts, 1969; Van Diggelen, Van Zeeland and Simons, 1972; Fujimoto and Seigmiller, 1970; Ashkenazi and Gartler, 1971) between cultured cells showing a deficiency in the enzyme HG-PRT (hypoxanthineguanine phosphoribosyl transferase) which confers resistance to the antimetabolite 8-azaguanine and wild-type cells which are sensitive to the drug. Most of the data are consistent with the idea that co-operation occurs by cell–cell contact, but some workers (Fujimoto and Seigmiller, 1970 and Ashkenazi and Gartler, 1971) have provided additional evidence that transfer may occur *via* mRNA or proteins, since HG-PRT negative cells can reacquire the enzyme and become sensitive to azaguanine after the addition of a 10,000 *g* supernate from wild-type cells or by the addition of conditioned media from dense wild-type cultures (Ashkenazi and Gartler, 1971).

The above phenomenon is well documented in the case of HG-PRT, has been observed for other phenotypes, and concerns the acquisition of drug sensitivity by resistant cells. On the other hand, there is relatively little information about transfer of resistance, in particular that to alkylating agents to sensitive cells. The only case appears to be that reported by Ujhazy (1969) who demonstrated that drug sensitivity of the Walker tumour decreased after it was passaged with Yoshida sarcoma cells that were resistant to a nitrogen mustard derivative TS-160 Spofa. Subsequently, Ujhazy *et al.* (1971) extended their observations and demonstrated that cytoplasmic fractions from L-asparaginase resistant Gardner lymphosarcoma and 5-fluorouracil resistant Ehrlich ascites carcinoma could decrease the sensitivity of sensitive cells, and that the effect was shown even when the cell homogenate was added *in vivo*. These workers also presented evidence to suggest that cytoplasmic fractions of the resistant cells were taken up by sensitive cells.

* On leave from: 1st Department of Pathological Anatomy, Semmelweis University, Budapest, Hungary

These phenomena thus resemble closely those attributed to metabolic co-operation, but in this case cell–cell contact appeared to be unnecessary.

Such phenomena could be of practical importance in tumour chemotherapy since they could increase the rate of development of resistance. Experiments were therefore designed to determine whether metabolic co-operation could play a role in the development of resistance of Yoshida rat sarcoma cells to methylene dimethanesulphonate (MDMS).

### MATERIALS AND METHODS

The MDMS-sensitive (YS) and MDMS-resistant (YR) Yoshida sarcoma cell lines were used, developed *in vivo* by Fox (1969) and isolated *in vitro* by Fox and Fox (1971). MDMS was prepared as described previously (Fox and Jackson, 1965).

The cells were cultured in suspension using Fischer's medium containing 20% horse serum (HOS). Treatment was carried out during the exponential phase ($1–2 \times 10^5$ cells/ml) of proliferation of the cells using drug dissolved in appropriate concentrations in physiological saline and sterilized by Millipore filtration. Plating in soft agar and determination of survival by extrapolation of growth curves were carried out according to the methods used in this laboratory as described previously (Fox and Fox, 1971). Autoradiography was carried out as described by Fox and Gilbert (1966).

The following experimental models were used:

*Co-culture of cells.*—We have previously demonstrated that YR cells are unable to form colonies in soft agar (Fox and Fox, 1971). Therefore co-culture experiments were undertaken with the expectation that any colonies produced had a high probability of being derived from sensitive cells. MDMS-sensitive Yoshida (YS) and MDMS-resistant Yoshida (YR) cells were therefore mixed with each other in various proportions, *i.e.* using 10–50–75–90% YS cells and 90–50–25–10% YR cells respectively. The cells of the 2 cell lines were cultured together for 24 hours. Following this 24-hour period of co-culture, the mixed cell population was exposed for 30 minutes to a range of doses of MDMS. The drug-containing medium was subsequently removed by centrifugation and the cells plated in normal growth medium in soft agar.

2. *Preparation of conditioned medium from YR and YS cells.*—Exponentially growing cultures of YS and YR cells were centrifuged and then suspended in fresh Fischer's medium containing 20% horse serum. After 2 days when the cell density had reached $\sim 4 \times 10^5$/ml the medium was removed by further centrifugation and the supernatant was used as conditioned medium from resistant cells (YRM) and sensitive cells (YSM). Various concentrations (10–100%) of YRM and YSM, diluted with Fischer's medium containing 20% horse serum, were used for culturing cells.

3. *Dialysis of medium of exponentially growing YR cells in the presence of YS cells.*—Since, in using the 2 methods just described, it was possible that some contamination of sensitive cells by resistant cells could occur, 2 ml of normal medium containing exponentially growing YR cells (approximately $4 \times 10^5$ cells/ml) were inoculated into a plastic dialysis bag previously sterilized by exposure to u.v. light (18 hours at dose rate $\sim$10 erg/sec) and the bag suspended in a bottle which contained exponentially growing YS (20 ml $\sim 5 \times 10^4$ cells/ml) cells in normal growth medium. After 24 hours the bag was removed, together with the YR cells, and the YS cells were harvested and subsequently treated with MDMS and plated in soft agar using normal medium.

### RESULTS

### *Co-culture of sensitive and resistant cells*

The results of the experiments using co-cultured cells are summarized in Fig. 1. When the mixture of 10% YS and 90% YR cells was exposed to MDMS the sensitivity of the cell mixture to MDMS decreased considerably. Smaller effects were seen with other mixtures of cells in other proportions but a decrease could still be seen in the case of the 25 : 75% and the 50 : 50% mixtures.

The plating efficiency of the cell mixtures was constant at 25–30% of the estimated number of sensitive cells, which is slightly lower than the plating efficiency

FIG. 1.—Dose response curves for Yoshida sensitive cells alone and for co-cultures of various proportions of resistant and sensitive cells plated in soft agar after treatment with MDMS. Co-cultures were grown for 24 hours before treatment with MDMS and plated in medium containing Fischer's medium and 20% horse serum (HOS). Under these conditions only sensitive cells are capable of forming colonies in soft agar. Results are mean of 2 experiments.

35–40% obtained when sensitive cells alone are plated. It has been previously shown that the resistant line will not form colonies in agar (Fox and Fox, 1971); therefore the observed colonies are probably derived from the sensitive line, suggesting the transfer of a " resistance factor ". This possibility has been further tested as follows:

In order to make sure whether both kinds of cells are present in the population after 24 hours, an exponentially growing culture of YR cells was incubated in the presence of $^3$HTdR (0·2 μCi/ml; 26 Ci/mM) for 24 hours. After this, a 50 : 50% mixture of labelled YR cells and unlabelled YS cells was prepared and the mixture cultured for 24 hours, and autoradiographs were subsequently made by the following method: After 24 hours of co-culture in suspension the cell pellet obtained by centrifugation (800 rev/min for 10 minutes) was washed twice with 0·9% saline, subjected to hypertonic treatment with 1% sodium citrate, then fixed and stained with acetic orcein. Autoradiographs were prepared as described by Fox and Gilbert (1966). Slides were exposed for 7 days and then the percentage of labelled cells was determined by scoring 5000 cells.

The cell population contained 60% YS cells and 40% YR cells—the proportion which would be expected, taking into account the very similar growth rate of the 2 cell lines reported before (Fox and Fox, 1971) and also found in our recent experiments. No phagocytosis of labelled cell particles by unlabelled cells was observed.

*Characterization of colonies surviving MDMS treatment after co-culture*

If the colonies surviving MDMS treatment after co-culture of sensitive and resistant cells are truly derived from sensitive cells then they should (a) retain their agar colony forming ability and (b) retain some evidence of transferred resistance. The following experiment was therefore performed:

A 10% sensitive: 90% resistant cell mixture was prepared and co-cultured in suspension for 24 hours. Aliquots of the mixture were then plated in agar with or without exposure to 40 μg/ml MDMS. After incubation for 10 days, colonies were isolated from control untreated mixed cultures S/RC and treated mixed cultures S/R40 and grown in suspension until enough cells were available for further testing. The sensitivity of these clones, *i.e.* S/RC and S/R40, to MDMS was compared with that of the original sensitive line YS and the original resistant line YR by the extrapolated growth curve

FIG. 2.—Effect of MDMS treatment on Yoshida S ○ and Yoshida R ▼ cells and on control (S/RC) ● and treated (S/R40) ▲ clones isolated after co-culture of 10% sensitive and 90% resistant cells in the same conditions as described in Fig. 1, where resistant cells are unable to form colonies in soft agar. Clones were cultured for 7 days after isolation before retesting. The 2 experiments were performed within 3 days of each other.

method (Fox and Fox, 1971) and by the agar cloning technique.

The dose response curves in Fig. 2 show that the clone S/RC was more resistant than the original sensitive line YS and that S/R40 was more resistant than either. S/R40 was, however, not as resistant as the original resistant line YR. Clones YS, S/RC and S/R40 all retained their ability to form colonies in agar but again no colonies were obtained when YR cells alone were plated. Although it can be argued from the results just presented (Fig. 1 and 2) that the YR cells may be rescued by some factor released by YS cells which enables them to form colonies in agar, this possibility has been discounted by experiments to be described in the next section.

*Transference of resistance by conditioned medium*

In the experiments described above it is possible that hybrid cells are produced, or that the effect is the result of metabolic co-operation resulting from cell–cell contact. To determine whether resistance could be conferred in the absence of resistant cells, we prepared conditioned medium as described in "Methods" from both sensitive (YSM) and resistant (YRM) cell cultures.

Using YRM and YSM conditioned medium, we initially determined whether the conditioned medium itself influenced the growth rate of YS or YR cells. The proliferation of both YS and YR cells cultured in 10 and 20% YRM or YSM was measured by counting the cell numbers on 5 subsequent days.

The cells of each type of culture showed a proliferation rate similar to those of the control cultures maintained in fresh normal medium (Table I). These data also demonstrate the similarity in growth rate of the 2 cell lines.

YR and YS cells were then cultured in conditioned medium for 24 hours before treatment (5–40 μg/ml MDMS for 30 minutes) and then plated in soft agar.

The dose response curves for YS cells cultured for 24 hours before treatment in 10–100% YRM are shown in Fig. 3. When 20% YRM was used the drug sensitivity decreased considerably, but at higher or lower concentrations the conditioned medium was not as efficient in protecting the YS cells against MDMS toxicity. As a control to these experiments and the co-culture experiment previously described, YR cells were cultured for 24 hours in YSM, then plated in soft agar and their colony forming efficiency determined. Growth in the presence of YSM for 24 hours did not confer colony forming ability on the

Table I.—*Growth of Yoshida Sensitive and Yoshida Resistant Cells over a 5-day Period in Fischer's Medium Plus 20% Horse Serum (HOS) in the Presence or Absence of 10% or 20% YRM or YSM Conditioned Medium, Compared with their Growth in Fischer's Medium Containing 20% Horse Serum (HOS) Alone*

| Medium | Cells | Cell number/ml on subsequent days ($\times 10^4$) 1 | 2 | 3 | 4 | 5 |
|---|---|---|---|---|---|---|
| 10% YSM | YS | 1 | 2·1 | 5·4 | 21 | 65 |
| 20% YSM | YS | 1 | 2·5 | 6·8 | 23 | 70 |
| 10% YRM | YS | 1 | 2·0 | 6·0 | 28 | 80 |
| 20% YRM | YS | 1 | 2·6 | 6·6 | 27 | 92 |
| Fischer's plus 20% HOS | YS | 1 | 2·4 | 6·9 | 30 | 90 |
| 10% YSM | YR | 1 | 2·4 | 4·8 | 16 | 52 |
| 20% YSM | YR | 1 | 2·0 | 4·4 | 16 | 70 |
| 10% YRM | YR | 1 | 2·4 | 5·6 | 18 | 80 |
| 20% YRM | YR | 1 | 2·8 | 6·0 | 19 | 92 |
| Fischer's plus 20% HOS | YR | 1 | 2·8 | 6·0 | 18 | 80 |

resistant cells: no colonies were seen in the 10 plates examined.

Fig. 3.—Effect of MDMS on Yoshida S cells cultured for 24 hours before treatment in the presence of various concentrations of YRM conditioned medium. Results are mean of 2 experiments.

*Effect of conditioned medium on the rate of breakdown of MDMS*

To determine whether there was a factor present in the YRM which was capable of accelerating the rate of drug breakdown, sensitive cells were treated with MDMS for 30 minutes in the presence of 20% YRM. Cells were then washed free of drug and conditioned medium and plated in soft agar. In addition, the effect of adding the YRM after treatment of sensitive cells with MDMS was determined to test whether protection could be afforded by post-treatment. Fig. 4 shows the dose response curves of the 2 experiments. There is a slight decrease of sensitivity in the low dose range when treatment was carried out in 20% YRM; otherwise the curves closely resemble the control curve. This slight decrease appears to reflect the multicomponent nature of the dose response curves as seen during development of resistance (Fig. 1 and 3), but we have no explanation at present for their complex pattern. Culture in YRM after treatment did not affect the dose response curve at all.

*Stability of transferred resistance*

In order to obtain information about the stability of the transferred resistance, YS cells, cultured for 24 hours in 20%

Fig. 4.—Effect of MDMS treatment on Yoshida S cells, treated or plated in soft agar in the presence of 20% YRM. ○ Control YS, × Cells plated in YRM after MDMS treatment in normal medium ● Cells exposed to YRM during 30-min treatment with MDMS.

Fig. 5.—Effect of MDMS treatment on Yoshida S cells, cultured for 24 hours in 20% YRM and after this for 24 hours in normal medium before exposure to MDMS. ○ Control, ● YS cells treated with YRM.

YRM, were cultured for a further 24 hours in normal Fischer's medium containing 20% horse serum before treatment with MDMS and plating in soft agar in normal medium.

The dose response curves shown in Fig. 5 indicate that there is still a decrease in sensitivity but that the cells have lost a significant proportion of the initial transferred resistance.

*Transfer of the " resistance factor " to sensitive cells through a semi-permeable membrane*

In an approach directed at establishing the chemical nature of the factor released by the drug resistant cells, we incubated YS cells under conditions in which they were separated from YR cells by a semi-permeable membrane as described.

The dose response curve in Fig. 6 shows a considerable decrease in sensitivity of YS cells incubated for 24 hours in the presence of YR cells. This result suggests that the " resistance factor " passes through the pores of the dialysis bag; therefore its molecular weight is lower than 10,000. The protective effect of the dialysed medium was approximately equal to the effect observed when 40% or 10% conditioned medium was used for culture of cells 24 hours before treatment. As a control to the above, in a separate experiment YS cells were cultured for 24 hours in the medium from inside the dialysis bag, from which the YR cells had been removed by centrifugation after they had been cultured inside the dialysis bag in the presence of sensitive cells for 24 hours. No protective effect of this " spent " medium was observed. This result suggests that the " resistance factor " diffuses through the dialysis membrane as rapidly as it is produced, and that it is equally rapidly absorbed by sensitive cells if they are present.

FIG. 6.—Effect of MDMS treatment on Yoshida S cells cultured for 24 hours in normal growth medium in which a dialysis bag containing a 2 ml culture of Yoshida R cells ($4 \times 10^5$ ml) was suspended. ● Control, *i.e.* YS cells cultured in normal medium before treatment, ○ YS cells cultured for 24 hours in normal medium together with YR cells but separated from them by a semi-permeable membrane.

### DISCUSSION

The behaviour of Yoshida cells sensitive to MDMS in response to exposure to cells resistant to MDMS closely resembles the behaviour of a number of HG-PRT-negative mammalian cell lines when either co-cultured with enzyme positive cells or exposed to extracts of, or conditioned medium from, enzyme positive cells. In the case of transference of MDMS resistance, cell–cell contact appears to be unnecessary and the factor is apparently transferred by a dialyzable fraction of medium from YR cells. A loss of protection afforded by the factor gradually occurs when the supply of it ceases. Similar observations have been reported by Ashkenazi and Gartler (1971), who suggested that either metabolic turnover of the factor occurred or that it was diluted out of the cells by division.

Two observations were made in the present study in relation to the stability of the transferred factor in sensitive cells. Firstly, in experiments in which colonies were isolated from mixed cultures some degree of resistance was retained over the 3-week period required for testing, as indicated by the reduced sensitivity shown by clones S/RC and S/R40 compared with that of the original YS cell line (Fig. 2). This finding suggests that the factor is relatively stable. The difference in sensitivity between clone S/RC and S/R40 suggests that there may be some enhancement or fixation of resistance due to drug selection. The degree of acquired resistance resulting from selection alone in the absence of co-culture with fully resistant cells is probably relatively small, since we have previously shown (Szende and Fox, 1973) that at least 3 successive exposures of YS cells to MDMS are necessary before significant resistance develops.

On the other hand, when sensitive cultures were exposed to conditioned medium YRM for 24 hours, then cultured in normal growth medium for 24 hours before exposure to MDMS, a significant proportion of the protection originally afforded by YRM was lost, cf. Fig. 5 and Fig. 3. In the case of co-culture experiments, therefore, resistance was apparently stable whereas when YRM was used approximately 50% of the protection conferred was lost during a 24-hour period of culture in normal growth medium. Several factors may be responsible for this difference. In the first case, clonal isolates were made after co-culture of sensitive and resistant cells whereas when YRM was used whole populations were studied. It is possible, therefore, that either there is a much more efficient transfer and retention of the factor when cell–cell contact occurs or that the efficiency of transfer of the factor is the same in both cases, but when whole populations are exposed only " competent " cells take up the factor. Competent cells may represent a small fraction of the total population, perhaps cells in a particular stage of the cycle, and they may divide more slowly after absorption of the factor, thus reducing their proportion relative to the total population.

The reduced effectiveness of high concentrations of conditioned medium may be due (a) to the presence of some toxic metabolites from resistant cells, as evidenced by a reduction in plating efficiency in cultures exposed to conditioned medium but no drug or (b) to the exhaustion of some growth promoting factors during the production of the conditioned medium.

In conclusion, therefore, we suggest that after the first treatment with a drug, a small proportion of the whole population survives which is resistant to this drug either by selection or as a result of mutation. Such cells release the proposed " resistance factor " into the intercellular fluid and it is then transferred to other drug sensitive cells. Thus, at the time of the second treatment a much higher number of cells survive and they, in addition to the resistance developed by absorption of the factor, may develop their own resistance due to further selection. In this way, resistance of the majority of the cell population could develop after a single treatment, as is observed in some instances after *in vivo* exposure of the Yoshida sarcoma to MDMS (Fox, 1969).

Extrapolation of this finding to the field of cancer chemotherapy suggests that the frequency of administration of certain drugs may be too high, and that less frequent drug administration, or more frequent alteration of the nature of the chemotherapeutic agent, could lead to a slowing down of the development of resistance of tumours to chemotherapeutic agents.

The work reported in this paper was undertaken during the tenure of a Research Training Fellowship awarded to B. Szende by the International Agency for Research on Cancer.

### REFERENCES

ASHKENAZI, Y. E. & GARTLER, S. M. (1971) A Study of Metabolic Co-operation using Human Mutant Fibroblasts. *Expl Cell Res.*, **64**, 9.

FOX, B. W. (1969) The Sensitivity of a Yoshida Sarcoma to Methylene Dimethane Sulphonate. *Int. J. Cancer*, **4**, 54.

FOX, M. & FOX, B. W. (1971) The Establishment of Cloned Cell Lines from Yoshida Sarcomas having Differential Sensibilities to Methylene Dimethane Sulphonate *in vivo* and Their Cross-sensitivity to X-rays, UV and Other Alkylating Agents. *Chem.-Biol. Interactions*, **4**, 363.

FOX, M. & GILBERT, C. W. (1966) Continuous Irradiation of a Murine Lymphoma Line P388 *in vitro*. *Int. J. Radiat. Biol.*, **11**, 339.

FOX, B. W. & JACKSON, H. (1965) *In vivo* Effects of Methylene Dimethane Sulphonate on Proliferating Cell Systems. *Br. J. Pharmac. Chemother.*, **24**, 24.

FUJIMOTO, W. F. & SEIGMILLER, J. E. (1970) Hypoxanthine--Guanine Phosphoribosyl-transferase (HG-PRT) Deficiency: Activity in Normal Mutant and Heterozygote Cultured Human Skin Fibroblasts. *Proc. natn. Acad. Sci., U.S.A.*, **65**, 577.

SUBAK-SHARPE, H., BÜRK, R. R. & PITTS, J. D. (1969) Metabolic Co-operation between Biochemically Marked Mammalian Cells in Tissue Culture. *J. Cell Sci.*, **4**, 353.

SZENDE, B. & FOX, M. (1973) The Establishment of MDMS-resistant Cloned Cell Lines from *in vitro* Cultured MDMS-sensitive Yoshida Sarcoma Cells. *Chem.-Biol. Interactions*, **6**, 19.

UJHAZY, V. (1969) Drug Resistance Study in a Mixed Nitrogen Mustard-sensitive/Resistant Tumour. *Neoplasma*, **16**, 467.

UJHAZY, V., JUZELA, S., KREMPASKY, V. & BOHUNICKA, E. (1971) Uptake of Drug-resistant Tumour Cell Fractions by Drug-sensitive Tumour Cells. *Neoplasma*, **18**, 627.

VAN DIGGELEN, M. C. E., VAN ZEELAND, A. A. & SIMONS, J. W. I. M. (1972) The Role of Metabolic Co-operation in Selection of Hypoxanthine Guanine Phosphoribosyl Transferase (HG-PRT) Mutants from Diploid Mammalian Cell Lines. *Mutation Res.*, **14**, 355.


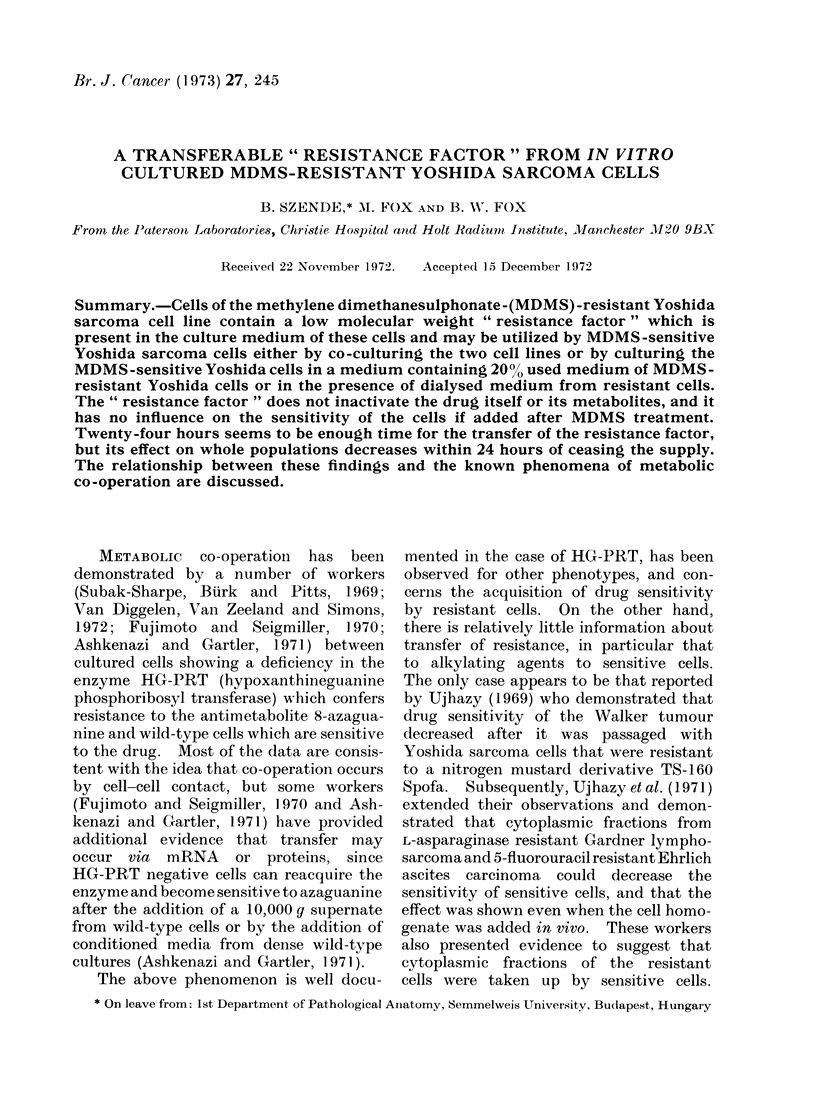

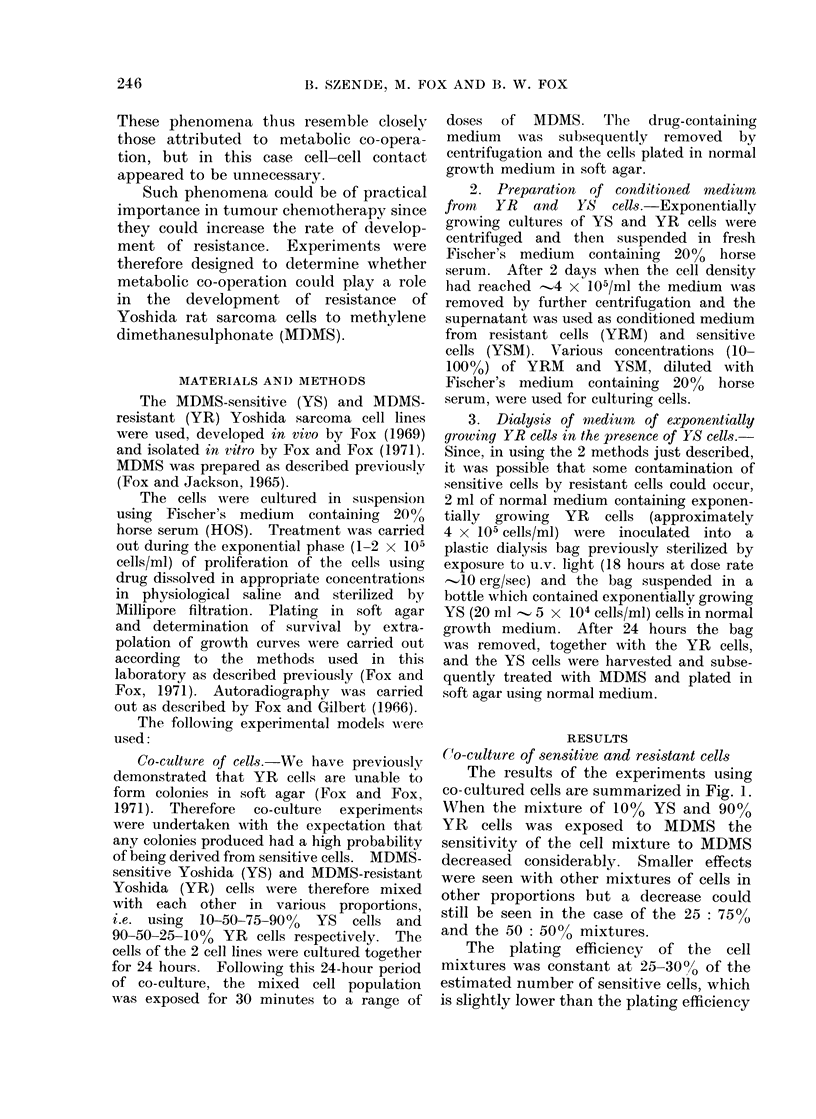

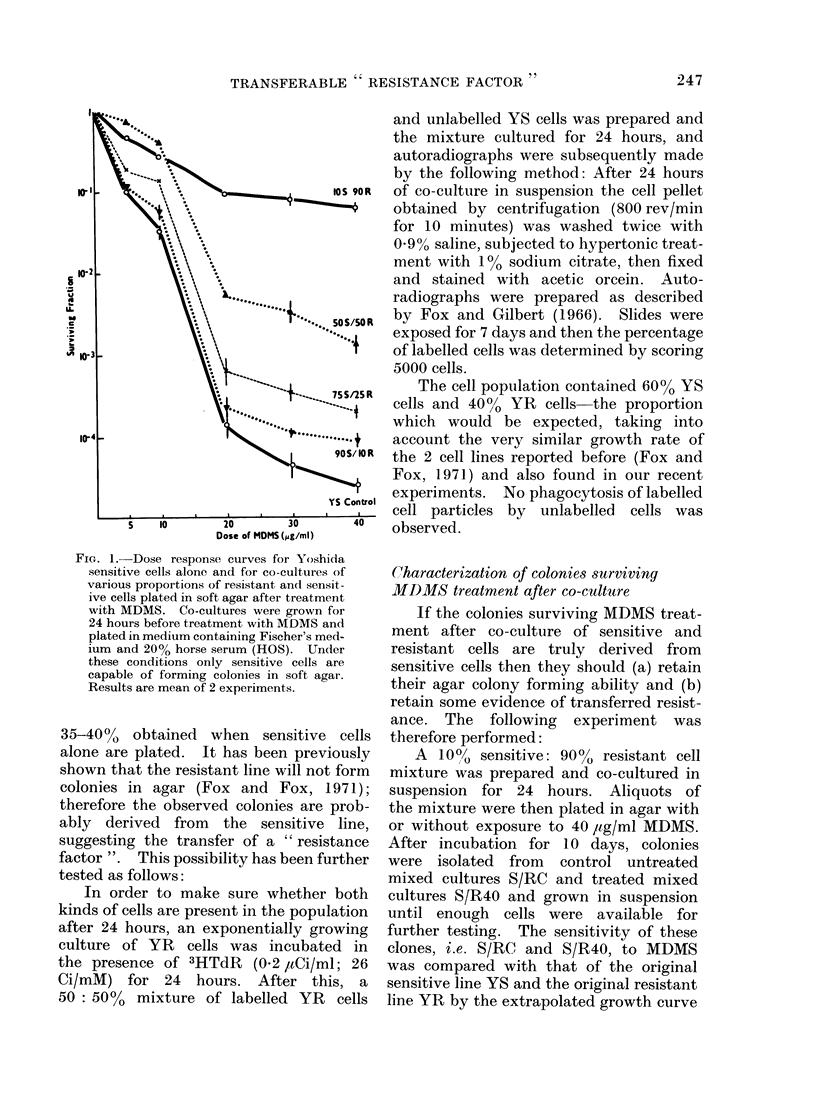

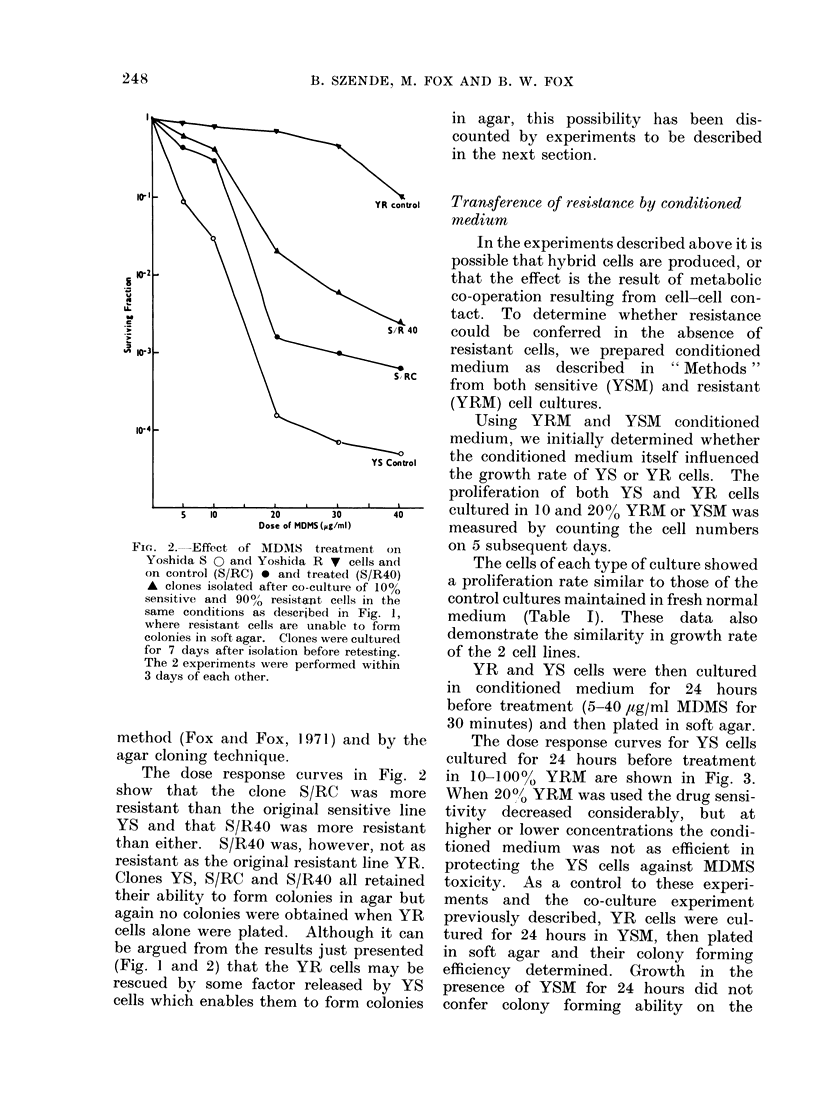

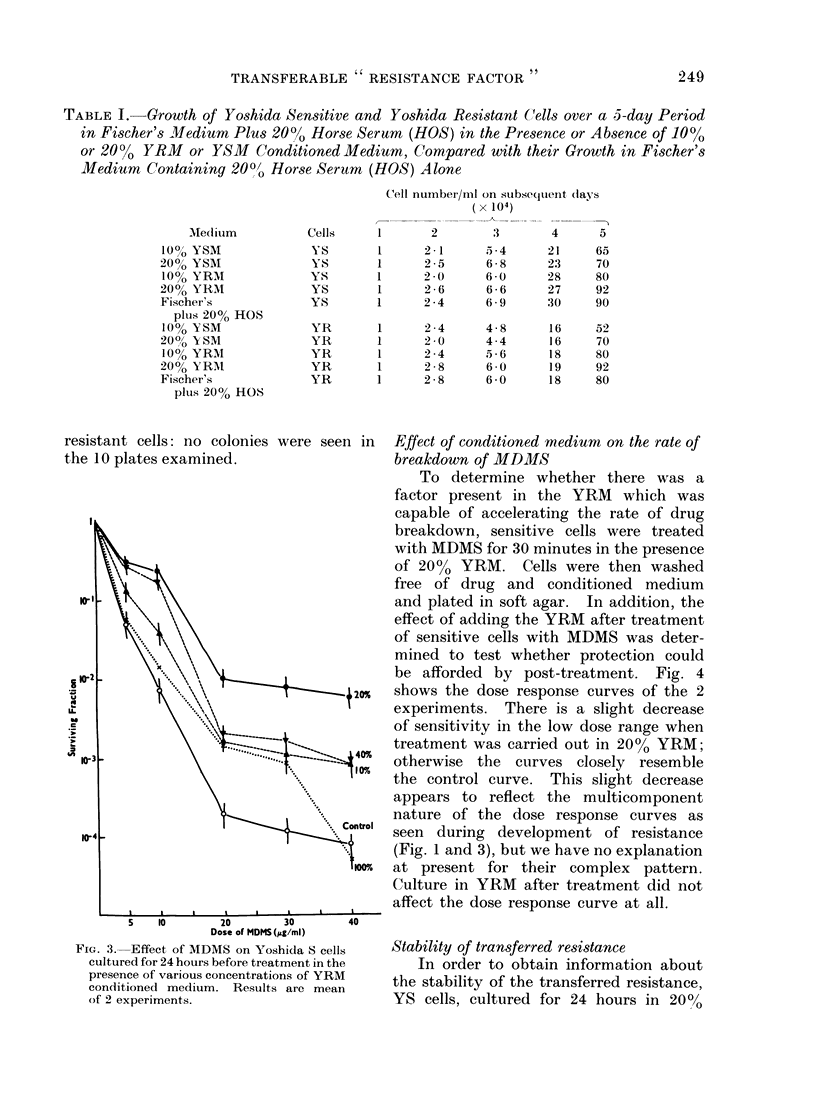

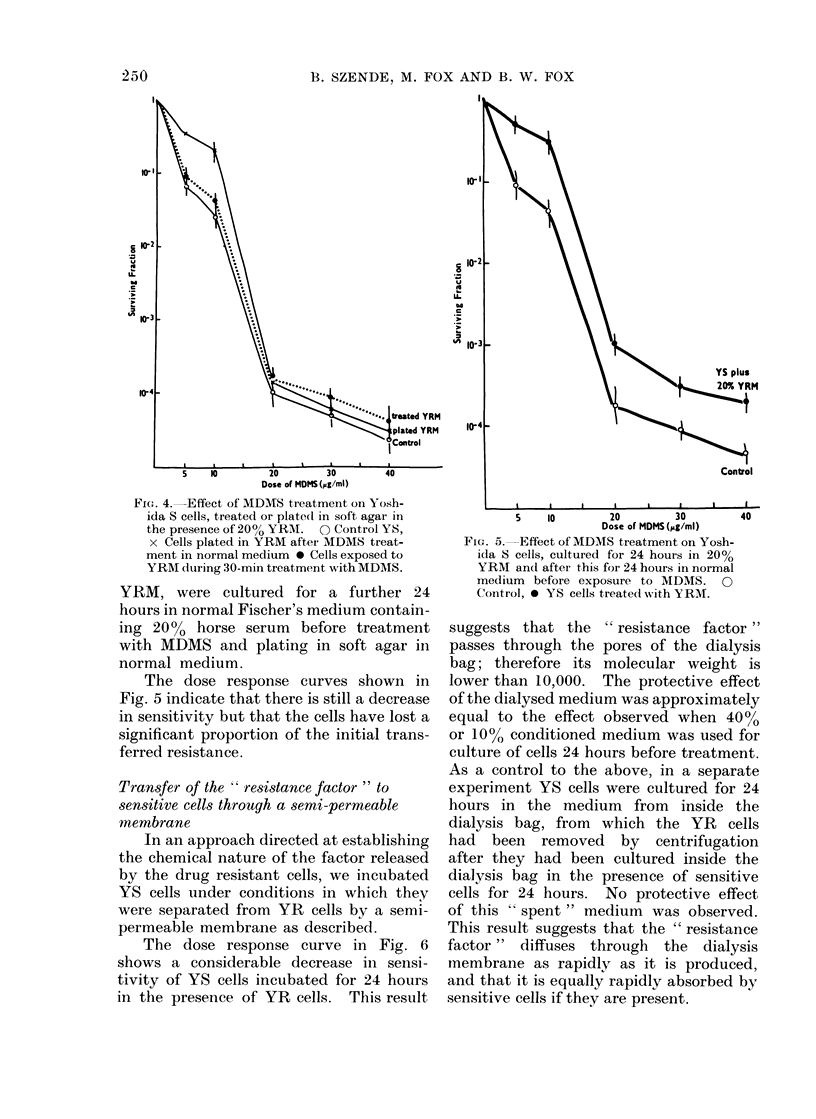

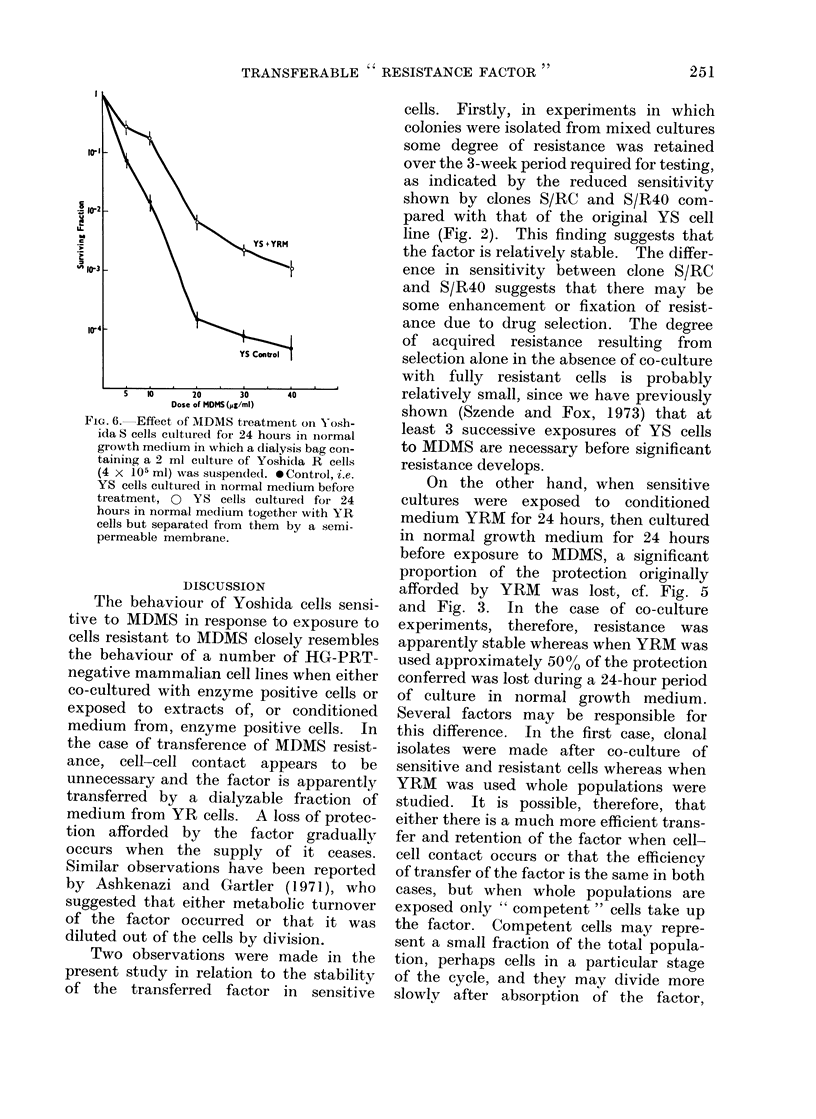

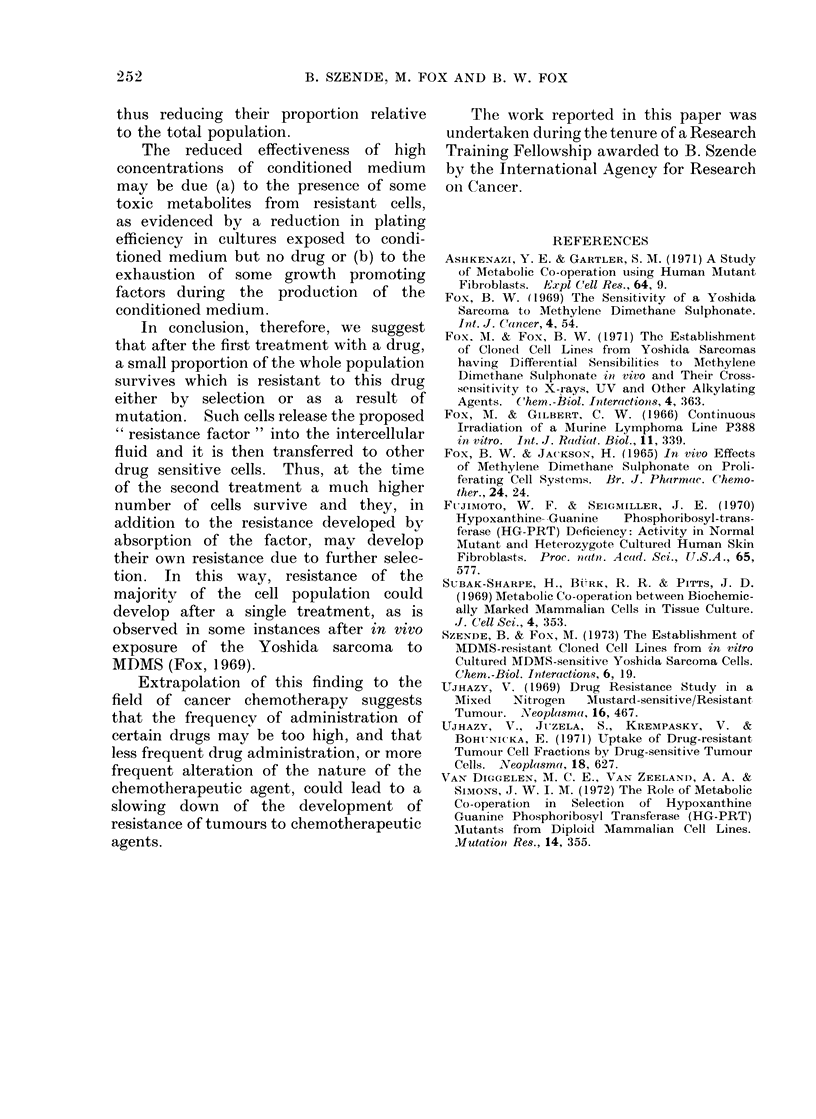


## References

[OCR_00733] Ashkenazi Y. E., Gartler S. M. (1971). A study of metabolic cooperation utilizing human mutant fibroblasts.. Exp Cell Res.

[OCR_00756] FOX B. W., JACKSON H. (1965). IN VIVO EFFECTS OF METHYLENE DIMETHANESULPHONATE ON PROLIFERATING CELL SYSTEMS.. Br J Pharmacol Chemother.

[OCR_00738] Fox B. W. (1969). The sensitivity of a Yoshida sarcoma to methylene dimethane sulphonate.. Int J Cancer.

[OCR_00743] Fox M., Fox B. W. (1972). The establishment of cloned cell lines from Yoshida sarcomas having differential sensitivities to methylene dimethane sulphonate in vivo and their cross-sensitivity to x-rays, UV and other alkylating agents.. Chem Biol Interact.

[OCR_00751] Fox M., Gilbert C. W. (1966). Continuous irradiation of a murine lymphoma line P388F in vitro.. Int J Radiat Biol Relat Stud Phys Chem Med.

[OCR_00770] Subak-Sharpe H., Bürk R. R., Pitts J. D. (1969). Metabolic co-operation between biochemically marked mammalian cells in tissue culture.. J Cell Sci.

[OCR_00776] Szende B., Fox M. (1973). The establishment of MDMS-resistant cloned cell lines from in vitro cultured, MDMS-sensitive, Yoshida sarcoma cells.. Chem Biol Interact.

[OCR_00782] Ujházy V. (1969). Drug resistance study in a mixed nitrogen mustard-sensitive-resistant tumour.. Neoplasma.

[OCR_00787] Ujhăzy V., Kuzela S., Krempasky V., Bohunickă E. (1971). Uptake of drug-resistant tumour cell fractions by drug-sensitive tumour cells.. Neoplasma.

